# Locally Informed Modeling to Predict Hospital and Intensive Care Unit Capacity During the COVID-19 Epidemic

**DOI:** 10.31486/toj.20.0073

**Published:** 2020

**Authors:** Daniel Fort, Leonardo Seoane, Graham D. Unis, Eboni G. Price-Haywood

**Affiliations:** ^1^ Center for Outcomes and Health Services Research, Ochsner Clinic Foundation, New Orleans, LA; ^2^ Division of Academics, Ochsner Clinic Foundation, New Orleans, LA; ^3^ The University of Queensland Faculty of Medicine, Ochsner Clinical School, New Orleans, LA

**Keywords:** *Coronavirus*, *COVID-19*, *critical care*, *hospitals*, *respiration–artificial*

## Abstract

**Background:** In the early phases of the 2019 novel coronavirus (COVID-19) pandemic, health system leaders faced the urgent task of translating the unknown into forecasting models for hospital capacity. Our study objective was to demonstrate the application of a practical, locally informed model to estimate the hospital capacity needed even though the community COVID-19 caseload was unknown.

**Methods:** We developed a susceptible-infected-recovered (SIR) model that was adopted from the University of Pennsylvania COVID-19 Hospital Impact Model for Epidemics and employed at 8 hospitals within Ochsner Health, the largest integrated delivery system in Louisiana, between March 16 and April 15, 2020. Intensive care unit (ICU) admissions of cases in the New Orleans area were used to estimate the community case load when testing was delayed.

**Results:** Initially, the observed ICU census trended near R_0_=2.0, whereas the ventilator census trended between R_0_=2.0 and 3.0. After implementing social distancing, both the ICU and ventilator capacity trended toward R_0_=1.3, while non-ICU medical/surgical beds trended toward R_0_=1.5. The model accurately predicted peak ICU (n=250) and hospital bed (n=487) usage by April 6, 2020. In response to model trends, Ochsner added 130 ICU beds across its hospitals by opening a new ICU and converting operating rooms and parts of emergency departments to ICU beds.

**Conclusion:** When disease testing is limited or results are delayed, ICU admissions data can inform SIR models of the rate of spread of COVID-19 in a community. Our model used various R_0_ plots to demonstrate an array of scenarios to guide planning for hospital and political leaders.

## INTRODUCTION

During the uncertainty of the early phase of the 2019 novel coronavirus (COVID-19) pandemic, hospitals and health system leaders faced the urgent task of translating the unknown into forecasting models of acute care, critical care, and ventilator capacity. A number of models emerged, with the University of Washington Institute for Health Metrics and Evaluation COVID-19 forecasting model garnering the attention of the federal government.^1^ A compilation of models was subsequently made available for quick access via the American Hospital Association.^2^ All of the models are based on assumptions about the current state of the disease, how quickly it spreads, and the degree to which interventions such as social distancing have been employed to slow the spread of infection. Many of the models are web-based tools that require local data entry and display outputs generated from background advanced statistical modeling. The primary challenge for many nonstatisticians and nonepidemiologists who must make key decisions for health systems, cities, or states is how to comprehend concepts of exponential growth and lag times and then to overlay these concepts with concrete bed or ventilator counts to predict future resource utilization.

Ochsner Health, the largest integrated delivery health system in Louisiana, USA, based in New Orleans, became one of the epicenters for the COVID-19 pandemic in early March 2020 following the annual Mardi Gras celebration. To help system leaders make operational decisions, we searched for a practical forecasting model that would be easy to translate locally without complex statistical modeling. We selected susceptible-infected-recovered (SIR) modeling given its ease of implementation. SIR models, however, can be challenging to use in the moment because they rely on known cases. In the early stages of the pandemic in the United States, testing was limited, turnover time for test results widely varied, and efforts toward standardized reporting of COVID-19 cases were delayed. In particular, standardized reporting for diagnosis codes and laboratory result codes had not yet been published, leaving healthcare systems with variable methods of manual case tracking. Notwithstanding these limitations, Ochsner leadership deemed the University of Pennsylvania COVID-19 Hospital Impact Model for Epidemics (CHIME)^3-4^ the most conceptually practical and informative tool.

This report describes the development of a simplified COVID-19 forecasting tool that was derived from the CHIME concepts, demonstrates the validity of our early modeling using real-world hospital census data, and shows how the tool was used to make operational decisions for a large health system in one of the COVID-19 epicenters.

## METHODS

### Study Population and Setting

Ochsner Health is the largest integrated delivery health system in the state of Louisiana, USA, and is headquartered in New Orleans. Ochsner owns or manages 40 hospitals and more than 100 health centers and urgent care centers, has almost 25,000 employees, and employs more than 1,300 physicians in more than 90 medical specialties and subspecialties. Ochsner uses Epic Systems electronic health records, and data for approximately 4.4 million patients from across the system are stored in the same Epic instance, allowing for robust, integrated reporting. This report is based on data collected from patients hospitalized between March 16, 2020 and April 7, 2020. The Ochsner institutional review board approved this study.

### Louisiana's Response to the COVID-19 Pandemic

The first presumptive case of COVID-19 in Louisiana was identified on March 9, 2020 in New Orleans. During the following week, patients with complications of COVID-19 infections began to be admitted to Ochsner hospitals and other area hospitals at a rate that would overwhelm system bed capacity in a few weeks. On March 16, 2020, the mayor of the city of New Orleans issued a social distancing proclamation. The governor of Louisiana issued a stay-at-home order on March 23, 2020, involving the closure of all nonessential businesses and educational institutions. Throughout this time, state officials worked closely with hospital executive leadership to estimate capacities for hospital beds, critical care units, and mechanical ventilation.

### COVID-19 Simple Susceptible-Infected-Recovered Model Construction

The SIR model compartmentalizes the population into the categories susceptible, infected, and recovered.^5^ On average, individuals develop symptoms of COVID-19 5 days after becoming infected.^6^ To estimate COVID-19 hospital utilization on any given day, we initially made several practical assumptions: (1) newly hospitalized patients represent 9% of the population, and a proportion of these patients require the intensive care unit (ICU) or the ICU with mechanical ventilation (3% and 2%, respectively); (2) hospital discharges reflect a range of patient care—from noncritical care to ICU care to ICU care with mechanical ventilation—with different average lengths of stay (7, 11, and 14 days, respectively); and (3) the hospital census for each level of care is a combination of carryover patients and newly admitted patients after adjusting the daily count for patients discharged. Refer to Table 1 for a summary of model parameters and Table 2 for the variables and formulas used to estimate the number of hospitalized patients according to the maximum required level of care.

**Table 1.** Initial Model Inputs

Parameter	Base Case Value	Data Source
Fixed parameters		
Greater New Orleans area population, n	1,262,888	Census track data
Ochsner Greater New Orleans market share, %	45	Ochsner executive leadership
Initial infected cases, n	10	Model assumption for imputation
Incubation, days	5	UPENN CHIME
Duration of symptoms, days	10	UPENN CHIME
Variable parameters		
R_0_	3	UPENN CHIME (doubling time of 6 days)
Hospitalization rate, %	9	Observed proportion based on Ochsner intensive care unit rate
Intensive care unit rate, %	3	UPENN CHIME
Mechanical ventilation rate, %	2	Local observed ventilator rate in Ochsner intensive care unit patients
Hospital length of stay, days	7	UPENN CHIME
Intensive care unit length of stay, days	11	Personal communication with University of Washington critical care
Mechanical ventilation length of stay, days	14	Local observed Ochsner length of stay

UPENN CHIME, University of Pennsylvania COVID-19 Hospital Impact Model for Epidemics.^4^

**Table 2.** Initial Model Definitions and Formulas Used for Population Estimates

Term	Definition
Hosp	Calculated number of hospitalized patients (including MEDSURG and ICU)
ICU	Calculated number of patients requiring care in the intensive care unit (including Vent and no Vent)
Vent	Calculated number of patients in the ICU requiring mechanical ventilation
MedSurg	Calculated number of patients requiring inpatient admission but not ICU care
Metro	Calculated totals for the Greater New Orleans Metropolitan Area
DC	Calculated number of patients discharged at the specified time point
**Input Variable**	**Definition**
R_0_	Basic reproduction number (R_0_=3; R_0_=2; R_0_=1.5 assuming social distancing)
β	Contacts per unit time
γ	1/mean recovery time = 1/(incubation + symptoms)
t	Time, days
S_t_	Total susceptible at time t
I_t_	Total infected at time t
New I_t_	Newly infected at t
R_t_	Total recovered at time t
New R_t_	Newly recovered at t
SX_t_	Calculated symptomatic at time t
INCub_t_	Calculated incubated at time t
**Population Estimate**	**Formula**
Susceptible today	S_today_ = S_yesterday_ – New I_today_
Infected today	I_today_ = I_yesterday_ + New I_today_ – New R_today_
Recovered today	R_today_ = R_yesterday_ + New R_today_
Symptomatic today	SX_today_ = number infected 5 days prior to time today = I_t-5_
Incubating today	INCub_today_ = I_today_ – SX_today_
Newly infected today	New I_today_ = (β * I_yesterday_ * S_yesterday_) / S_initial_
Newly recovered today	New R_today_ = γ * I_yesterday_
New Metro Hosp	Number newly infected 5 days prior * 0.09
New Metro ICU	Number newly infected 5 days prior * 0.032 – New Metro Vent
New Metro Vent	Number newly infected 5 days prior * 0.023
New Metro Hosp_DC	Number hospitalized 7 days prior / 7
New Metro ICU_DC	Number patients in ICU – no Vent 11 days prior / 11
New Metro Vent_DC	Number patients in ICU 14 days prior / 14
Total Metro Cases today	Total Metro Cases_yesterday_ + New Metro Hosp_today_ + New Metro ICU_today_
Total Metro inpatients today	Total Metro Hosp_today_ + Total Metro ICU_today_ + Total Metro Vent_today_
Total Metro Hosp today	Total Metro Hosp_yesterday_ + New Metro Hosp_today_ – New Metro Hosp_DC_today_
Total Metro ICU today	Total Metro ICU_yesterday_ + New Metro ICU_today_ + New Metro Vent_today_ – New Metro ICU_DC_today_ – New Metro Vent_DC_today_
Total Metro Vent today	Total Metro Vent_yesterday_ + New Metro Vent_today_ – New Metro Vent_DC_today_
Market share = 45%	MedSurg_today_ = Total Hosp_today_ * 0.45 ICU_today_ = Total ICU_today_ * 0.45 Vent_today_ = Total Vent_today_ * 0.45

Using the latest New Orleans census track population data (n=1,262,888) and a random assumption of 10 initial cases, we calculated imputations of the number of susceptible, infected, and recovered individuals in the community over time (Supplement, available on request by emailing ocjournal@ochsner.org). We constructed a simple SIR epidemic model using Excel (Microsoft Corp.) in which the formulas in Table 2 were embedded to allow for rapid visualization and investigation of parameter changes. We initially assumed an R_0_=3.0 (doubling time of 6 days, based on the CHIME model specifications) on March 18, 2020, the last date prior to the original model publication on March 19, with the 4 days of initially observed data starting March 16.^4^ Two major changes had to be incorporated during the first 3 weeks of model observation: the mayor's social distancing proclamation and the governor's stay-at-home order. Each of these social interventions was assumed to effectively reduce infectivity to an R_0_=2.0 on March 16 and to R_0_=1.5 or 1.3 on March 23, with full effects of those changes trailing by approximately 10 days. Branching forecasts were carried forward until enough ICU census data were available to determine the true observed course. These branching forecasts allowed hospital executives to make operational plans based on multiple possible scenarios.

The March 23 version of the model also reflected expanded interest in bed utilization beyond ICU beds. Forecasting medical/surgical (MEDSURG) noncritical care demand began after an initial internal observation of a 3:1 MEDSURG to ICU admission rate. The imputed non-ICU hospitalization rate was 9%, assuming a 3% ICU hospitalization rate. Subsequent observations demonstrated 2:1 MEDSURG:ICU census.

### COVID-19 Actual Hospital Census Data

Starting March 16, 2020, staff of the Ochsner Pulmonary Critical Care Department began collecting census data on manually identified COVID-19–confirmed cases and persons under investigation for each ICU and emergency department–boarded admission across the entire Ochsner system. The homogenous presentation of critically ill patients with COVID-19 allowed us to reliably identify patients with COVID-19 infection at a time when the lack of testing did not allow the accurate calculation of spread in the community. Patients were also categorized as to whether they received mechanical ventilation.

On March 19, 2020, we used 4 days of manually compiled census data to fit an initial SIR epidemic model that we then tracked to evaluate model fidelity during the following 3 weeks.

To better understand the arrival and flow of patients with COVID-19, a report was created to capture COVID-19–confirmed patients by site, unit, and ventilation status. Each unit was mapped to a level of care: emergency department, ICU, MEDSURG, or other. The other designation included departments such as labor and delivery, psychiatry, and behavioral health where care was assumed to be unrelated to COVID-19 status; those data were not modeled. Patients on mechanical ventilation were identified with the additional status of Vent. By tracking patients across each daily census file in which they appeared, the number of days at each level of care were compiled for each admission, as well as key care transitions such as initiation of mechanical ventilation, care upgrade to ICU, or stepdown to MEDSURG.

### Qualitative Model Validation

The model was started with an initial 10 infected individuals and run forward. A starting point in terms of a known calendar date was identified by matching a modeled number of total ICU patients to the observed manual census. The model was deemed qualitatively valid and useful if the 10 days of subsequently observed census continued to track the model forecast, thereby allowing adequate time for initial expansion of ICU bed capacity.

We constructed 2 sets of model forecasts. The first was the original 2-week forecast (March 16 to March 31, 2020) based on the initial 4 days of observed census data paired with the subsequently observed ICU census data and expanded ICU capacity. The second model forecast was an updated 2-week forecast (March 23 to April 7, 2020) that accounted for expanded social interventions.

## RESULTS

### Simple Susceptible-Infected-Recovered Forecast

**Figure 1.**
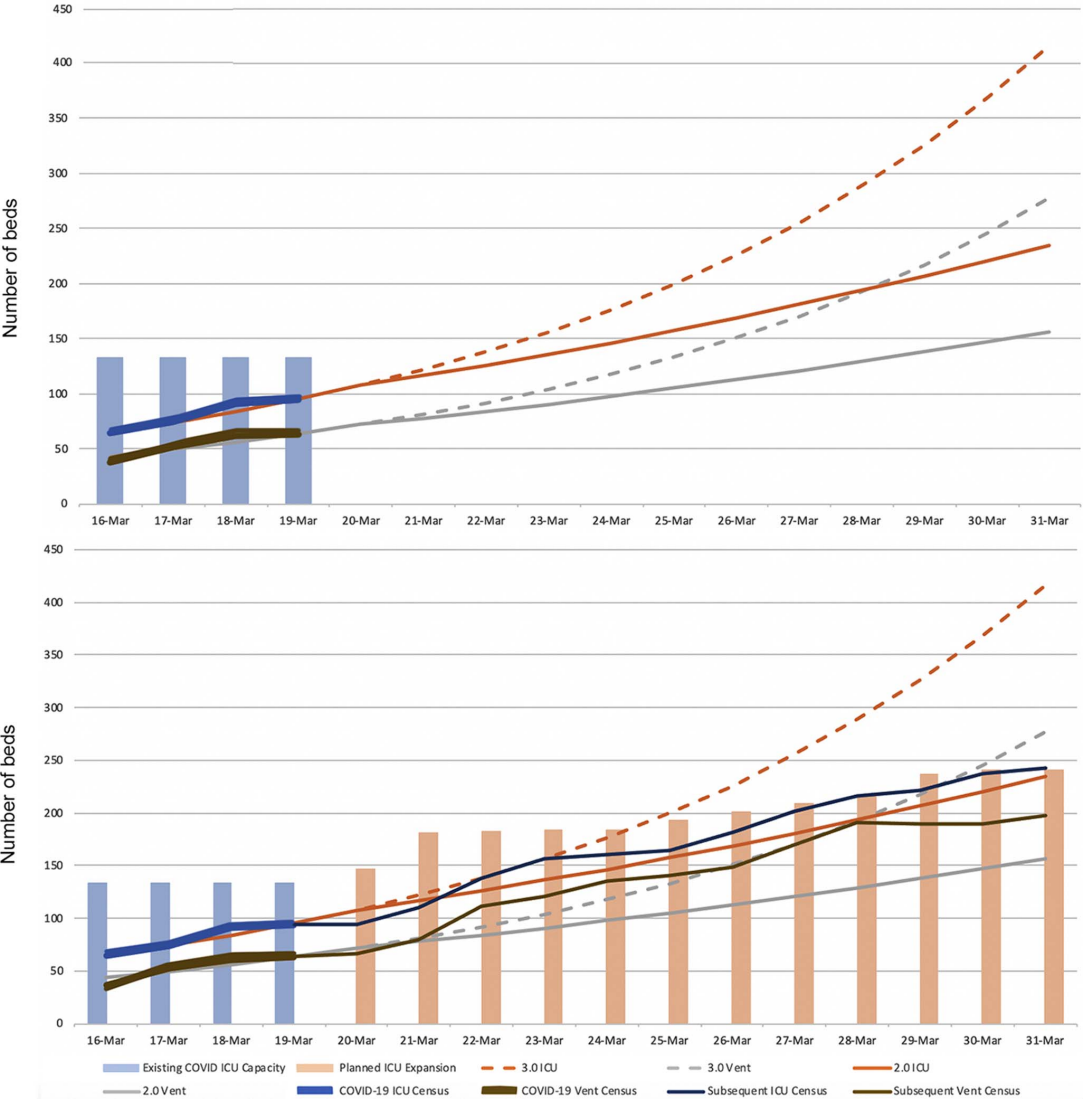
**Initial susceptible-infected-recovered model projection with subsequent 10 days of COVID-19 intensive care unit (ICU) census and expanded system ICU capacity, demonstrating early effects of social distancing on March 16, 2020.** COVID-19, 2019 novel coronavirus; Vent, mechanical ventilation.

**Figure 2.**
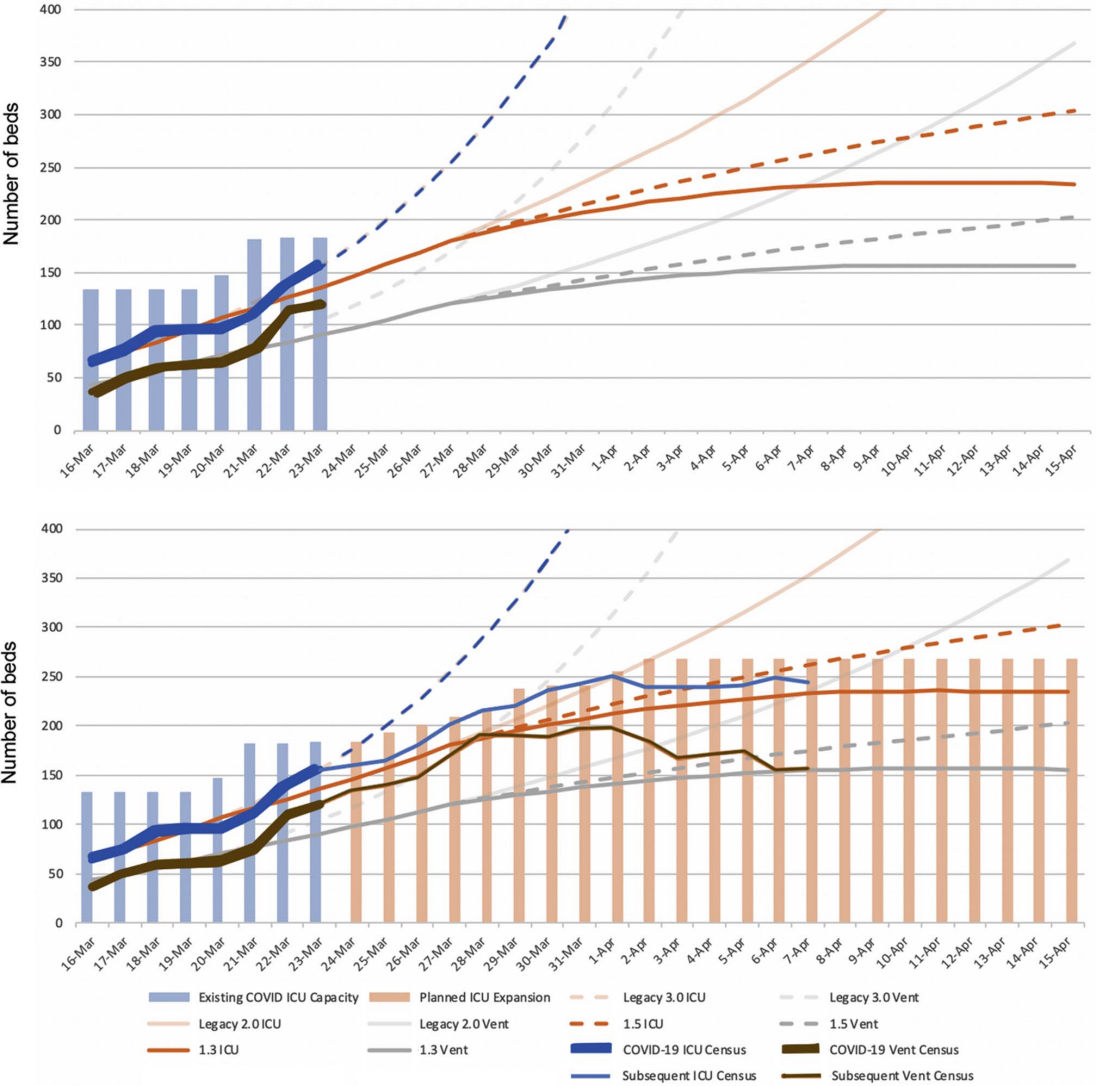
**Susceptible-infected-recovered model projection of potential reduced infectivity because of Louisiana governor's stay-at-home order starting March 23, 2020, with projected intensive care unit (ICU) capacity expansion and the actual observed ICU and mechanical ventilator (Vent) census.** COVID-19, 2019 novel coronavirus.

**Figure 3.**
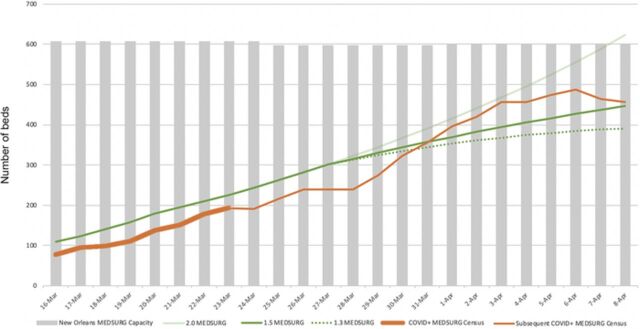
**Susceptible-infected-recovered model projection of medical/surgical (MEDSURG) bed demand with potential reduced infectivity (R_0_=1.5, 1.3) because of Louisiana governor's stay-at-home order starting March 23, 2020, with actual observed MEDSURG census and system MEDSURG bed capacity.** COVID+, positive for 2019 novel coronavirus.

Figure 1 displays the initial forecast and subsequent observed ICU/ventilator census and expanded bed capacity. The observed COVID-19 ICU census appeared to trend near an R_0_=2.0, whereas the COVID-19 ventilator census trended between R_0_=2.0 and 3.0. Figure 2 displays the follow-up forecast and subsequent observed ICU/ventilator census and expanded bed capacity after real-time hospital data were acquired. Figure 3 displays the prediction curve for MEDSURG noncritical care and the observed subsequent census. By the end of the observation period, both the COVID-19 ICU and ventilator capacity appeared to be trending toward an R_0_=1.3, while MEDSURG trended toward R_0_=1.5 subsequent to implementation of social distancing interventions.

Ochsner Health used data modeling to quantify and determine the time frame needed to expand capacity for ICU beds and redeploy staff. Overall, Ochsner added 84 ICU beds at its main tertiary facility in the first 3 weeks after New Orleans became an epicenter for COVID-19. ICU bed capacity was also increased by 46 beds at community hospitals by opening a new ICU and converting operating rooms and parts of emergency departments to ICU beds. The model provided support for physician and administrative leaders’ decisions around canceling elective surgeries; not accepting transfers from outside the region; and redeploying surgeons, anesthesiologists, and nurses to COVID-19–related clinical duties. The model accurately predicted peak ICU (n=250) and MEDSURG (n=487) bed needs by April 6, 2020. The model also helped the health system leaders make workforce decisions, such as how many agency nurses would be needed, and supply chain decisions regarding the purchase of pharmaceuticals, ventilators, and other supplies. Finally, long-term modeling led to the decision to expand the critical care tower at the main tertiary hospital by building 102 new ICU beds by the end of June 2020.

## DISCUSSION

Numerous mathematical models have emerged to predict the future of the COVID-19 pandemic in the United States and globally.^2^ The most effective use of these models is to estimate the effect of various interventions for reducing overall disease burden.^5,7-8^ Early in the pandemic, uncertainty about the actual number of cases, availability and/or accuracy of diagnostic testing, differences in the reported reproductive number, heterogeneity of subpopulations, and the yet-to-be-seen effects of social distancing generated a high level of urgency to find a practical model that could be used on the frontlines of healthcare. We developed a modified version of the University of Pennsylvania CHIME model^3-4^ that can be used by hospital executives and political leaders to make short- and long-term operational decisions about capacity, supply chain, and ventilator needs.

On the Gulf Coast of the United States, leaders are accustomed to emergency planning because of experience with hurricane-related natural disasters. Hurricane forecasts use spaghetti plots to show potential hurricane paths. Likewise, our model used various R_0_ plots to demonstrate an array of utilization scenarios in a manner familiar to our healthcare and political leaders. Because we were also plotting real-time hospital bed and ICU bed use along different infection rates, we were able to provide timely evidence of the impact of our regional nonpharmacologic mitigation measures on the local R_0_. Our model provided a means for regional hospitals in the New Orleans area to come together to assess MEDSURG bed capacity, ICU bed capacity, and ventilator needs. Area health systems agreed to provide daily data to our modelers and to regularly meet to coordinate emergency response. The health systems agreed to not have any one hospital go on diversion. Instead, admissions were coordinated to transfer patients and resources within and between the health systems to prevent any one hospital from being overwhelmed. The model helped inform the utilization and resource needs for the entire region. As mitigation measures are eased, our model will provide valuable data for our leaders to make decisions. We will continue to track our R_0_ after mitigation measures are relaxed and can advise local leaders if it demonstrates a concerning rise over time.

Our study has several limitations. Our use of ICU census as the single source of truth for documented cases of COVID-19 when we did not have reliable testing was one of the innovations of the model and proved to be very reliable. However, ICU admissions may not represent the true number of patients needing ICU care when ICU units reach capacity and patients are admitted to nontraditional ICU areas or transferred to hospitals outside the region. We accounted for this limitation by manually evaluating every admission and communicating daily with critical care physicians across our hospitals to account for all ICU patients. During peak local COVID-19 infectivity–late March and early April 2020–patients were transferred between hospitals but always within the region. Ochsner is the principal referral hospital for the region; therefore, some COVID-19 admissions were transferred in and may have resulted in an overestimation of community spread. However, the number of these patients was limited because Ochsner stopped accepting transfers from outside the region during this time frame.

Our study is also limited by changes in management of the disease over time. Initially, the critical care teams intubated patients with respiratory failure early to close the circuit and protect healthcare workers from droplets. As understanding of the treatment of COVID-19 evolved, our critical care teams adopted evidence-based practices used for other forms of hypoxemic respiratory failure, including high-flow nasal cannula, continuous positive airway pressure, and bilevel positive airway pressure with good success in keeping patients off the ventilator and as an adjunct to removing patients from the ventilator. As a result, our models soon demonstrated a much lower need for mechanical ventilators. Finally, our model represents the epidemiology of COVID-19 spread in the New Orleans area where we had a large inoculating event (Mardi Gras) and may not be representative of other regions.

## CONCLUSION

Our simplified SIR model offers leaders a practical approach to epidemic modeling that assesses utilization needs based on service areas of a given hospital. When testing is limited or results are lagging, ICU admissions data can be used to inform SIR models of the rate of spread of COVID-19 in a community.

## Notes

This article meets the Accreditation Council for Graduate Medical Education and the American Board of Medical Specialties Maintenance of Certification competencies for Medical Knowledge, Systems-Based Practice, and Practice-Based Learning and Improvement.
